# DNA deformability defines sequence-dependent capture of *E. coli* gyrase

**DOI:** 10.21203/rs.3.rs-7265879/v1

**Published:** 2025-08-18

**Authors:** Matthew L. Baker, Haley R. Johnson, Ryan A. Eckerty, Jonathan M. Fogg, Silvia L. Summers, Marlène Vayssières, Nils Marechal, Valérie Lamour, Wilma K. Olson, Lynn Zechiedrich

**Affiliations:** 1 Department of Biochemistry & Molecular Biology, University of Texas Health Sciences Center at Houston, Houston, TX USA;; 2 Graduate Program in Quantitative & Computational Biosciences, Baylor College of Medicine, Houston, TX, USA;; 3 Department of Molecular Virology & Microbiology, Baylor College of Medicine, Houston, TX, USA;; 4 School of Biological Sciences, Georgia Institute of Technology, Atlanta, GA, USA;; 5 Verna and Marrs McLean Department of Biochemistry & Pharmacology, Baylor College of Medicine, Houston, TX, USA;; 6 Université de Strasbourg, Centre National de la Recherche Scientifique (CNRS), Institut national de la Recherche Médicale (INSERM), Institut de Génétique et de Biologie Moléculaire et Cellulaire (IGBMC), UMR 7104- UMR-S 1258, F-67400 Illkirch, France;; 7 Department of Integrated Structural Biology, IGBMC, Illkirch, France;; 8 Hôpitaux Universitaires de Strasbourg, Strasbourg, France;; 9 Department of Chemistry & Chemical Biology, Center for Quantitative Biology, Rutgers, The State University of New Jersey, Piscataway, NJ 08854, USA

**Keywords:** DNA supercoiling, DNA minicircle, DNA site recognition, DNA base-pair step deformability, Type II topoisomerase

## Abstract

Bacterial gyrase, unique among type II topoisomerases, introduces negative supercoils into DNA. Mechanistic details of gyrase still must be elucidated because of the complexity of the process and the difficulty in visualizing it. Specifically, the interplay among base sequence, local DNA deformability, and global DNA topology for gyrase site selection is unclear. To understand how gyrase interacts with DNA and selects a site of action, we created an *ad hoc* shape-based recognition methodology to ascertain DNA sequence from cryogenic electron microscopy densities as a string of purines and pyrimidines, which we conclusively matched to the DNA in our two previous structures of negatively supercoiled DNA bound to *E. coli* gyrase. The DNA helices to be cleaved by gyrase (the Gate or G-segments) in both structures mapped to each side of a palindrome in the minicircle, with the DNA (relative to the enzyme) in opposite orientations. For one structure, the G-segment sequence was among the most flexible in the minicircle, facilitating the observed bend in the DNA. The flanking sequence was highly inflexible, which presumably prevented wrapping about the β-pinwheel of gyrase. For the other structure, in which the negatively supercoiled minicircle wrapped a positive supercoil around a β-pinwheel of gyrase, the G-segment contained base-pair steps of only average deformability. This work highlights how base sequence and local deformability around the site of action expedite DNA wrapping to facilitate the negative supercoiling activity of gyrase. It further demonstrates the utility of identifying protein-interacting DNA sequences from cryo-EM structures.

## Introduction

Essential and ubiquitous, type II topoisomerases pass DNA helices through each other to modulate DNA supercoiling, unlink DNA catenanes, and untie DNA knots^1–5^. Several steps are required: the enzymes bind DNA, cleave the DNA (becoming transiently covalently attached to newly formed ends), pass an intact DNA helix through the break and then religate the DNA backbone^6^. Typically, type II topoisomerases will act with high processivity, catalyzing several cycles of cleavage, strand passage, and religation before dissociating from the DNA^7,8^. Type II topoisomerases require a divalent cation to cleave and religate. ATP binding supports DNA strand passage but is not required for the remaining steps; ATP hydrolysis is required for enzyme turnover. Unique among type II topoisomerases, DNA gyrase can strand-pass without ATP, at least *in vitro*^9^. New insights into the molecular mechanisms of topoisomerases continue to emerge, yet there remains much we do not understand.

**Fig. 1.**
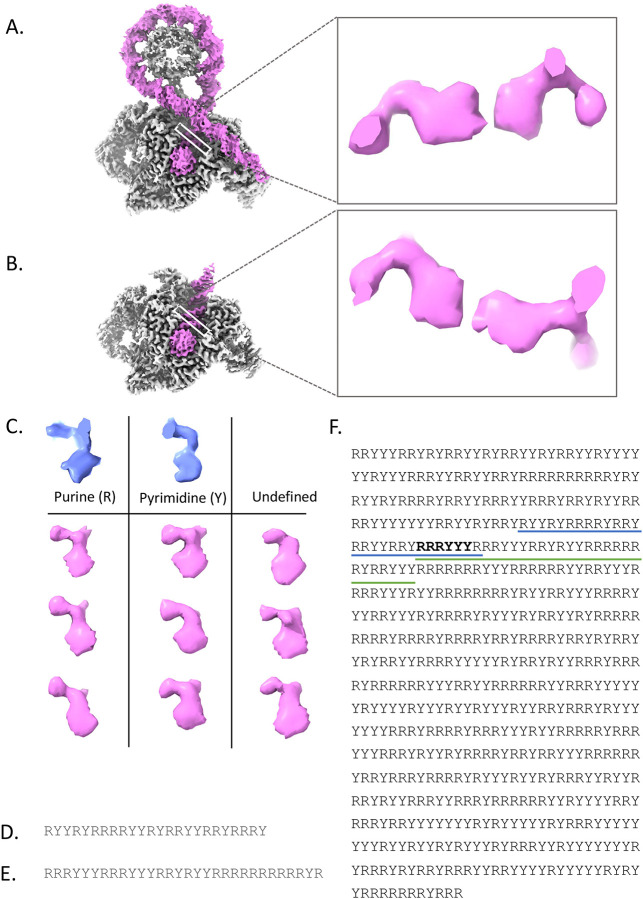
Negatively supercoiled minicircle DNA bound to gyrase. The cryoEM reconstruction of the DNA-wrapped gyrase (A) or the not-wrapped gyrase (B) is shown with the protein colored in grey and the dsDNA colored in violet. In both (A) and (B) a zoomed in view of an extracted base pair from the G-segment DNA (bound to the gyrase) is shown. All nucleotides bound to gyrase at the G-segment were computationally extracted (violet). Shown in (C), individual bases were compared amongst each other and against purine and pyrimidine simulated density maps (~3 Å resolution, cornflower blue). The sorted bases were assigned as purines (R), pyrimidines (Y), or undefined. From the sorting results and enforcing standard base pairing rules, a R/Y profile was constructed for the G-segment in wrapped and not-wrapped gyrase complexes, shown in (D) and (E), respectively. These profiles were used to search an R/Y profile of the 601-bp minicircle (F). The blue and green underlined sequence represents the alignment of the R/Y profile for the wrapped structure and not-wrapped structure, respectively. The bolded residues highlight the palindrome sequence.

We recently reported two near-atomic (2.9 and 3.0 Å) resolution structures of a catalytically inactive *E. coli* DNA gyrase mutant (GyrA^Y122F^) bound to a 601 base pair (bp) negatively supercoiled DNA minicircle using single-particle electron cryo-microscopy (cryoEM) (Fig. 1A,B)^10^. In one structure, which we refer to as the “wrapped structure” (Fig. 1A), a 118 bp segment of the negatively supercoiled minicircle looped around the β-pinwheel domain of GyrA to form a positively supercoiled crossover (against the negative supercoiling in the minicircle), simultaneously providing both the DNA segment to be cleaved (the G-segment) and the DNA segment to be transferred (the transfer- or “T-” segment) through the cleaved G-segment. The second reconstructed density map (Fig. 1B) showed a 30 bp G-segment of the minicircle^10^, which we call the “not-wrapped structure.”

In our previous attempts to model the DNA, several putative purine/pyrimidine sequences spanning the well-defined bases were generated and aligned to the minicircle sequence, but a unique mapping of the DNA bases from the density to the sequence could not be determined^10^. ModelAngelo^11^, an automated, machine learning-based cryoEM model building tool, failed to yield an unambiguous model/sequence correspondence^10^. We had speculated that this failure to obtain a sequence might derive from averaging of gyrase bound to multiple different sites along the minicircle^10^. Therefore, like for other recent protein-DNA complexes imaged by cryoEM, such as condensin^12^, the origin recognition complex^13^, MukBEF^14^, FtsK^15^, and many more, the DNA in the two gyrase structures was modeled as a generic adenine-thymine repeat^10^.

Not strictly site-specific, type II topoisomerases cleave linearized, relaxed, or positively or negatively supercoiled double-stranded DNA with the same reproducible sequence patterns^16–19^. Although gyrase must be able to bind and act at many different DNA sequences to perform its essential roles, certain base-pair steps^20,21^ would more readily accommodate the sharp bend in the G-segment. In addition, the DNA must be flexible enough to wrap the β-pinwheel. To determine which sequence(s) in the 601 bp minicircle might accommodate these specific binding requirements, we developed and utilized an *ad hoc* shape recognition and correlation methodology to classify individual DNA bases as purine- or pyrimidine-like from the cryoEM density maps. The resulting purine-pyrimidine sequences were then easily mapped to the known DNA minicircle sequence. The G-segment bound by gyrase for both structures mapped to an overlapping region of the minicircle containing a short (6 bp) palindrome but in opposite orientations.

To probe DNA sequence-dependent deformability^20,21^, we developed a DNA sequence analysis tool, which revealed that both DNA G-segments and the sequence that wrapped the β-pinwheel domain of gyrase had above average base-pair-step-dependent deformability. The sequences flanking the G-segment in the structure that did not wrap around gyrase had much lower than average sequence-dependent deformability. Additionally, structural analysis of the DNAs^22^ revealed several base pairs distorted from B-form and significant conformational transitions in the gyrase-bound DNA double helix. These findings support a new model for the molecular mechanism of DNA gyrase that features supercoiled DNA as a key player.

## Results

### Existing tools fail to reveal DNA sequence

To determine which DNA sequence(s) *E. coli* gyrase bound in the 601 bp negatively supercoiled minicircle, we initially analyzed the DNA densities with various cryoEM modeling software, including Phenix^23^ and CryoREAD^24^. The map_to_model procedure^25^ in Phenix was unable to produce a model for the well-resolved portions of the DNA and contained incorrect base pairing, which prevented successful mapping of the DNA back to the known minicircle sequence. CryoREAD, a deep-learning-based nucleotide modeling tool for cryoEM density maps at resolutions of 10 Å or lower, produced models with incorrect base pairing, incompatible chain directions, and broken DNA backbones (Fig. S1). Because the training sets for CryoREAD only contained linear (relaxed) DNA, it was possible that the negative supercoiling in our densities prevented success of existing methods.

### Shapes of DNA bases

For over two decades, shape-based methods have been used to identify structural features and build models for proteins in subnanometer resolution cryoEM density maps^26–34^. We reasoned that this approach should work for distinguishing DNA bases as well. Therefore, we examined individually extracted bases from the cryoEM density maps and classified them by shape similarity to template purine and pyrimidine structures at similar resolutions (Fig. 1C and Fig. S2). We generated the average template shapes via two different methods with the same results. First, we manually examined the shape of each individual base in the best resolved sections, which were those in the G-segment, in both of our structures. We noticed geometrical features (ultimately deemed oval-like and bending) that separated the bases into two distinct shapes. At the same time, we examined various purine and pyrimidine maps and models from the Protein Data Bank (PDB) at atomic level resolution (<3 Å or better). The models were blurred to the approximate resolution of our cryoEM density maps (2.5–3.2 Å) using e2pdb2mrc.py from EMAN2. From the atomic level data, we saw that purines are wider and have a narrower groove than pyrimidines (Fig. 1C).

Importantly, because we only examined the bases (excluding potentially confounding information from the sugars and phosphates), the relative translations of each base to its base pair or among base-pair steps are inconsequential. Therefore, bending and twisting resulting from the negative supercoiling of the minicircles does not interfere with the process.

### Base Hunting

We first manually assigned each base as most like purine (R) or pyrimidine (Y) without regard to the base-paired strand. We then checked that each base pair contains one of each. This approach resulted in a simple DNA pattern for each DNA strand—a series of consecutive annotations where each base is labeled R or Y—teased directly from the density maps (Fig. 1D,E). It is important to note that through this step, we did not consider the known DNA sequence. In the final step (Fig. 1F), we converted the known minicircle sequence to an R/Y string and matched the two R/Y patterns we had generated. We then substituted the actual minicircle sequence back into the matched pattern to reveal the sequence in the maps.

**Fig. 2.**
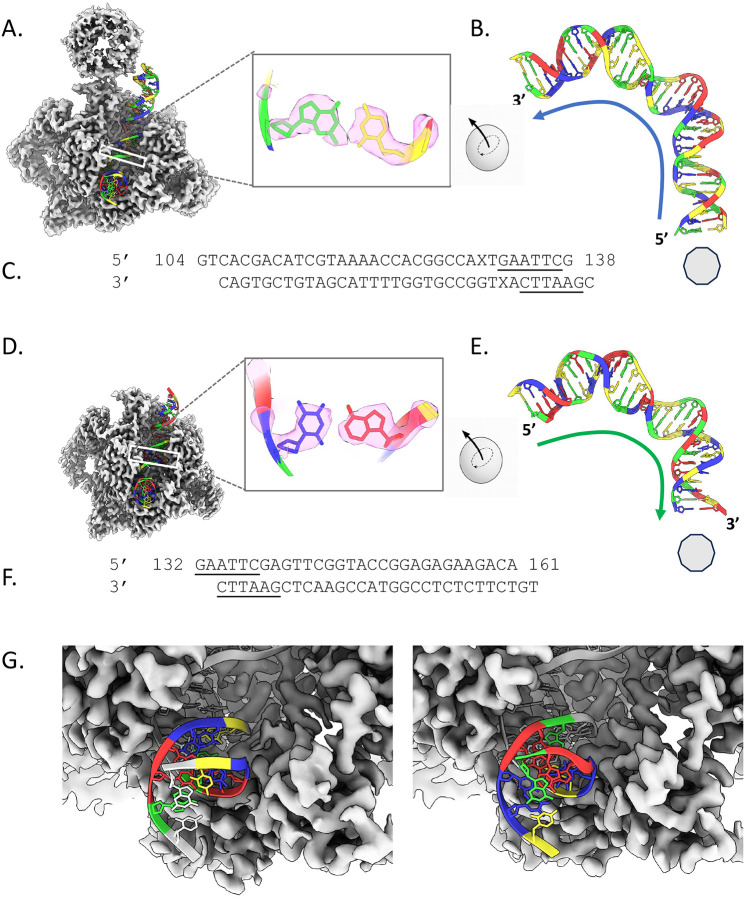
Modeling negatively supercoiled minicircle DNA G-segments. From the R/Y profile alignments, a complete sequence model was constructed for the G-segment DNA in the wrapped and not-wrapped complexes shown in (A) and (D), respectively. Zoomed in views of a single base pair fit to the density are boxed; green is guanine, red is adenine, blue is thymine, and yellow cytosine. This color scheme is used throughout the manuscript. In (B) and (E), models for the G-segments for the wrapped and not-wrapped structures are shown. The extracted models were rotated such that the palindrome sequence is displayed in the upper left. The rotation is indicated by a tracing of a geodesic arc corresponding to a ~109.5 ° angular displacement (solid angle = ~3.82 steradians). The direction of the G-segment DNA-strand containing the GAATTC-palindrome relative to β-pinwheel (depicted as a light grey polygon) is indicated by the arrows (blue arrow denotes the wrapped and green arrow the not-wrapped G-segments). The sequence corresponding to the modeled G-segment of the wrapped and not-wrapped is shown in (C) and (F), respectively. The underlined regions indicate the palindrome. In (G), a zoomed in view of the palindrome and its position in the G-segment relative to the wrapped (left) and not-wrapped (right) gyrase complexes are shown.

In examining both gyrase-DNA cryoEM density maps, we easily established the DNA sequence register in the structure for the bent G-segment DNAs in both models (Fig. 2, Tables 1–2). In the model of the negatively supercoiled minicircle wrapped around the β-hairpin of gyrase, the wrapped conformation (Fig. 2A–C), a DNA density profile was generated for the well-resolved G-segment DNA corresponding to positions 92–115 in Chain E and positions 4–27 in Chain F of the previously deposited model (PDBID:8QDX; we maintained this nomenclature in our newly deposited structures). 22/24 bases in our *ad hoc*-generated DNA pattern (R/Y) matched a pattern of bases at positions 112–135 (as assigned in^10^) in the minicircle DNA (Table 1). Density corresponding to base pairs at positions 122 and 130 were indistinguishable as R vs. Y. Nonetheless, with this alignment, we were able to extend the DNA sequence to structure assignment to cover base pairs 109–138 of the minicircle DNA at the G-segment. These base pairs contained nucleotides that are not as well resolved but still were sufficient for subsequent modeling.

For the other structure (gyrase bound only to the G-segment DNA, the not-wrapped conformation) (Fig. 2D–F), our shape modeling similarly yielded a clear assignment for 28/30 bp, corresponding to base positions 132–161 of the minicircle DNA (Table 2). The base pairs that could not be assigned corresponded to positions 10 and 30 in Chain E (and positions 20 and 1 in Chain F, respectively in 8QQS). The probability of matching either of the two gyrase-minicircle DNA structures by random chance would be 1.79 × 10^−5^ and 4.34 × 10^−7^ for the wrapped and not-wrapped structures, respectively, lending confidence to our findings.

### Gyrase binds a short palindrome from both sides of the DNA helix

We used standard model building and refinement tools to replace the A/T DNA segments used previously^10^ with the correct DNA sequence. In the final models, the overall structure of the DNA for both G-segments, despite opposite orientations and mostly distinct base compositions, is nearly identical (RMSD <1 Å) (Fig. S2). The DNA undergoes bending and unwinding of the type first seen in complexes with the TATA-box binding protein^35,36^. Both G-segment deformations accompany the partial intercalation of isoleucine 174 from the two GyrA subunits at sites on DNA separated by 12 bp and the transformation from the B to the A double-helical form of eight intervening residues in contact with both GyrA and GyrB (Fig. 4). There is a 7 bp overlap between the two G-segment structures that resides in the exact same position relative to gyrase—the “front” of the DNA-binding domains of GyrA/B (Fig. 2G, Fig. S2). This overlap includes the palindromic sequence—5’-GAATTC-3’ (Fig. 2G, Fig. S2). As such, the corresponding base pairs can be placed in the same physical location of the DNA-binding domain no matter the directionality of the DNA. Gyrase has been previously reported to bind to repeat palindromic sequences^37^, but never before, that we can find, to a single palindrome.

### Structurally interpreting established gyrase motif

A DNA sequencing approach, called Topo-Seq^38,39^, provides precise single-nucleotide resolution mapping of topoisomerase cleavage sites. Analysis of the thousands of gyrase sites observed by Topo-Seq revealed a 130 bp “gyrase motif.” This motif consists of three regions—an initial 47 bp region with periodic distribution of G/C content, a 36 bp central region, and another 47 bp G/C periodic region. The DNA sequence in our wrapped complex is approximately 50 % G/C, which is evenly distributed along the sequence. The sequence in our not-wrapped complex is A/T-rich and contains long stretches with only A/T. If DNA wrapping by gyrase is facilitated by phased G/C content^38,40^, this difference may explain why one sequence wrapped and the other one did not in our structures.

In our wrapped structure^10^, the DNA wraps 48 bp around the β-pinwheel and then passes through the central region, which is the G-segment, over 35 bp. Thus, our sequence lacked the second periodic region. In a later publication that utilized a linear DNA molecule containing the naturally occurring strong gyrase site, MuSGS, to generate a wrapped gyrase-DNA structure^41^, the second region was also seen wrapped around the β-pinwheel. MuSGS closely matches the Topo-seq determined gyrase cleavage motif, and contains two periodic regions, therefore allowing wrapping about both β-pinwheels.

There were other striking structural deviations in the DNA wrapped around the pinwheel in our structure that may help determine sequence preference for gyrase. There are stretches of localized DNA unwinding from B- to A- and TA-forms (Fig. 4). GC-rich sequences show a greater propensity to undergo B-A transitions^42^ and exhibit more A-like character, *e.g*., lower values in intrinsic twist, in high-resolution structures^21^. Thus, a previously unrecognized function of the G/C periodicity observed in the gyrase motif^38^ may be to enable these transitions.

Comparison of the identified gyrase binding sites to the position weight matrix of the gyrase cleavage motif^32^ gives a numerical score that describes how well a sequence fits the motif, with a positive score indicating that the sequence fits the motif. When considering the entire 130 bp gyrase motif identified by Topo-seq, there were no positive scores within ± 5 bp of the center of the G-segment (because we used a catalytically inactive mutant gyrase, we will not assume exact cleavage positions), for either the wrapped or the not-wrapped structures. When comparing the sequences we observed to bind to gyrase in our structures (one β-pinwheel and the G-segment for the wrapped structure and only the G-segment for the not-wrapped structure) to the corresponding regions of the Topo-Seq gyrase motif, however, there were several positive (though low) scores within ± 5 bp of the center of the G-segment of both structures (Table S2). This motif analysis, given the data we have, therefore fails to fully explain why gyrase bound the sites it did on our supercoiled minicircle.

### Importance of DNA flexibility for gyrase binding

DNA sequence strongly dictates which DNA segments can be deformed, bent, compressed, and moved as each base pair step has an inherent deformability that is influenced by the base pairs around the base pair steps^20,21^. Unrelated sequences can have similar deformability, and similar sequences can have vastly different deformability. It seems reasonable that not every DNA sequence can bend to conform to the requirements (~72 °) for the G-segment binding to gyrase, bending that may be stabilized by the insertion of the aforementioned enzyme isoleucine side chains into the DNA helix^43^. It seems even less likely that just any DNA sequence could accommodate the tight wrap seen around the β-pinwheel. Because the distance between the end of the gyrase-bound G-segment and the start of the β-pinwheel wrap is so short (~30 Å), these two segments must be contiguous. Therefore, a DNA site simultaneously must have a bendable G-segment and a contiguous segment deformable enough to wrap the β-pinwheel.

To identify flexible regions within the minicircle, we employed a sliding-window analysis of DNA sequence-dependent deformability^20,21^ based on base-pair step parameters. In this approach, a window of fixed length *k* (*i.e*., a *k*-mer) is passed across the sequence to calculate the average deformability score of all steps within that *k*-mer (see Fig. S3), analogous to classical hydropathy plots used in protein sequence analysis^44^. This *k*-mer deformability score reflects how intrinsically deformable the base pairs within the segment are based on their local sequence composition, with higher scores indicating greater capacity for bending, unwinding, or wrapping. It is important to recognize that these deformability scores are not the actual deformability of a *k*-mer of DNA but instead are a description of how deformable the individual base pair steps within the *k*-mer are when averaged over *k* length. It is unclear how the deformability of a DNA sequence changes as the sequence in question grows, but certainly this averaging dampens the extremes on both ends of the scale.

Applying our *k*-means averaging method to the 601 bp minicircle (Fig. 4) revealed that the deformability score of the base pair steps within the 30 bp G-segment of the not-wrapped structure is in the top 2 % most flexible of all possible 30-mers in the 601 bp minicircle (Fig. 4b). Additionally, the center of the G-segment of the not-wrapped structure contains the single most deformable base pair step—TA within the tetramer GTAC. These extremely flexible pyrimidine-purine steps are seen as sharply bending hinges at DNA-protein binding sites^20^.

Contrasted to the high flexibility of the not-wrapped G-segment, the adjacent region of the minicircle that should have wrapped the β-pinwheel in the not-wrapped structure has an extremely low 49-mer deformability score, falling in the bottom 2.6 % of all 49-mers in the minicircle sequence. This rigidity, in the region of the minicircle that would be expected to wrap the β-pinwheel of gyrase, provides an explanation as to why this sequence did not wrap. In the wrapped structure, the average deformability of the 35 bp that form the G-segment falls in the top 47 % most deformable of all 35-mers in the minicircle whereas the sequence in the wrap is in the top 20 % most flexible by average deformability score (Fig. 4b). This analysis revealed that gyrase is attracted by two elements—G-segment bending, which is promoted by base-pair step deformability, and flexibility of the DNA that will wrap the β-pinwheel.

## Discussion

We developed and utilized a shape-based method to directly determine purine/pyrimidine sequences from cryoEM density maps. We used these sequences to query the known DNA minicircle sequence and precisely determine the sequence of DNA bound to gyrase and successfully construct more complete gyrase-DNA models.

Because it relies solely on the shape of individual bases, our method is agnostic to the form (B or non-B), structure, or topology of DNA. Indeed, as noted above, the central section of the G-segment in type II topoisomerase complexes is not B-, but A-form^38^, yet we were nonetheless able to correctly determine the sequence of the G-segment. This approach may even be suitable for analyzing double-stranded RNA, and perhaps eventually single-stranded RNA, although the confidence gained from base pairing rules is an important aspect of our *ad hoc* method. A limitation of this procedure is that it requires relatively high resolution of individual bases; we estimate better than ~3 Å based upon where in the density models we could no longer distinguish R from Y.

DNA gyrase wraps DNA in a positive wrap to impart directionality on strand passage^10,45^. The localized unwinding of DNA seen in the structures of both the G-segment and pinwheel-wrapped segment facilitate this wrapping. The insertion of stretches of locally underwound A-DNA within a B-DNA helix yields a right-handed superhelix in much the same way that the insertion of overwound base-pair steps yields a left-handed superhelix^46^ (Fig. S4).

Some of the earliest insights into the mechanistic basis of the sequence preference of DNA gyrase resulted from the discovery of naturally occurring strong gyrase sites (SGS). The best studied of these, MuSGS, is essential for the efficient replication of the bacteriophage Mu^47^. The ability of the MuSGS to promote gyrase binding and activity was proposed to be related to the phased anisotropic bending sites within the sequence, facilitating DNA wrapping around the GyrA C-terminal domain (CTD)^40^. It should be noted that the 601 bp minicircle sequence we used previously^10^ did not contain any known strong gyrase sites. Thus, the requisite flexibility may be provided by the negative supercoiling in the minicircle. The subsequent structure of gyrase bound to a linear MuSGS-containing DNA shows the same features of localized DNA unwinding^41^. Thus, both sequence and supercoiling make contributions to DNA wrapping.

The DNA palindrome, GAATTC, is the only sequence common to both the wrapped and not-wrapped gyrase complexes, suggesting that it may be a key determinant for where gyrase binds the 601 bp minicircle. An important caveat to interpreting these data, however, is that the minicircle was attached via streptavidin-biotin to the cryoEM affinity grid. How this immobilization technique might impact the DNA sequences gyrase binds is unknown.

The palindromic sequence found in both of our gyrase-minicircle structures is also recognized by the restriction enzyme EcoRI. Many restriction enzyme sites are palindromic, which allows the dimeric enzymes to bind the two half-sites symmetrically, facilitating sequence recognition^48^. Restriction sites exhibit very high sequence discrimination with typically 10^6^-fold preference in *k*_*cat*_*/K*_*m*_ for cleaving their given site compared to other sites differing by a little as 1 bp^49^. This sequence preference is significantly higher than for gyrase, but the molecular mechanism of EcoRI may provide insight into why gyrase binds this palindrome.

EcoRI recognizes its restriction site through a mixture of direct and indirect readout^50^. The palindrome in the crystal structure of EcoRI-bound DNA is kinked at the center^51^. This DNA bending is a contributor to sequence recognition through indirect readout. In comparison, the palindrome in the gyrase structures is not in the bent section. The palindrome at the EcoRI site also allows for “crosstalk” between the two active sites of the enzyme^52^. Direct readout of DNA sequence by EcoRI and other sequence-specific enzymes involves specific contacts between the protein and the bases via the major and minor grooves. In the gyrase structures, the protein contacts the sugar phosphate backbone of the palindrome rather than the bases, suggesting that indirect readout may be more important for gyrase.

The sequence used for the first crystal structure of B-DNA, the “Drew-Dickerson dodecamer,” also contained an EcoRI site at the center^53–56^. As a result, the GAATTC palindromic sequence has become well-studied. Electrophoresis^57^ and NMR^58^ studies suggest that the sequence may be intrinsically curved in solution, even though it is not in the crystal structure^55^. Intrinsic DNA curvature localizes at superhelical apices^59^. A short stretch of naturally unwound GC-rich base-pair steps could certainly localize to apices (Fig. S4). Were gyrase to preferentially bind at superhelical apices, the enzyme, therefore, would be brought into the vicinity of the palindrome.

Previous studies that have investigated sequence specificity of gyrase have looked for cleavage sites; however, the relationship between DNA cleavage and strand passage is not fully understood. For one thing, it is unclear whether more cleavage leads to more strand passage. That cleavage occurs without ATP suggests that the two activities—cleavage and strand passage—can be decoupled. We suggest a model in which the subtle protein and DNA movements that accompany gyrase-mediated cleavage provide added flexibility to perform a conformational selection for wrapping. If the DNA adjacent to the G-segment is flexible enough to wrap, the T-segment becomes positioned to enable strand passage, as in our wrapped structure. If the adjacent region is not flexible enough, as in our not-wrapped structure, gyrase will religate the G-segment and (eventually, depending upon the off-rate) continue a search for a different G-segment with adjacent DNA that would allow the wrapped DNA crossover conformation required for both G- and T-segment binding. In other words, not every DNA sequence adjacent to the G-segment will accommodate the tight positive wrap^20,21^. Sutormin and coworkers found that the gyrase consensus sequence they discovered surprisingly inhibited gyrase activity when incorporated on a plasmid^38^. They hypothesized that the high affinity of gyrase for the consensus sequence may actually inhibit strand passage. Thus, the enzyme may become stalled at the cleavage step. The complete catalytic cycle requires DNA cleavage, wrapping about the CTD, strand passage and finally unwrapping to allow for selection of a new T-segment or dissociation from the DNA.

In summary, this study provides a tool for interpreting DNA sequence in 3D cryoEM structures, a tool to evaluate DNA deformability, and insight into the mystery of how DNA gyrase choreographs a complex and essential DNA dance.

## Methods

### Data processing

The density maps and models for the wrapped (EMD-18605, PDBID:8QDX) and not-wrapped (EMDB-18603, PDBID: 8QQS) gyrase were downloaded, segmented, and split into “gyrase” or “DNA” maps using the coordinate data as a reference with the “Color by Zone” tool in ChimeraX (V1.7.1) with a radius of 10 Å. Initial evaluation of the DNA revealed a stretch of well-resolved base pairs corresponding to bases 89–118 on chain E (nomenclature from ref.^10^ and the PDB) and 1–30 on chain F in the wrapped structure; with bases 92–115 in Chain E (and the corresponding bases in Chain F) having the highest local resolution. Well-resolved DNA density was also observed in the same location in the not-wrapped DNA-gyrase map, corresponding to the entirety of chains E and F of the deposited model (bases 1–30 in both chains). This region was in the DNA-binding domain of GyrA/B and appeared to be the G-segment DNA.

### Modeling with Phenix and CryoREAD

While previous modeling tools did not produce reliable DNA models^10^, we initially attempted to build models for the well-resolved portions of the DNA using Phenix map_to_model and CryoREAD. DNA from the two deposited maps was isolated and segmented using ChimeraX^60^. Density maps containing only the full segmented DNA and a truncated version consisting of only the well-resolved portion of the DNA at the DNA/gyrase interface for both DNA-gyrase reconstructions was submitted to the CryoREAD^24^ webserver (https://em.kiharalab.org/algorithm/CryoREAD, accessed March/April, 2024). For each submitted map, the resolution parameter was set at 3 Å and CryoREAD was run with and without setting an explicit contour value, and with and without inputting the minicircle DNA sequence. Additionally, Phenix’s map_to_model (Phenix v1.19), which can generate a model without the user providing a protein or DNA sequence, was run with default options from command line on a Linux workstation. The resulting models for both the wrapped and not-wrapped DNA from both modeling tools yielded non-Watson-Crick base pairing, broken chains, directional inconsistencies, failure to fit the DNA model, and incompatibility with the known minicircle DNA sequence (Supplementary Fig. 1B).

### Base hunting

As CryoREAD, Phenix, and ModelAngelo did not produce meaningful base assignments, we adopted our own identification and modeling strategy that focuses on categorizing base shapes as purine or pyrimidine. Using the DNA models from the deposited structures (AT-only models) as a template, every base in the well-resolved portions of the DNA maps was segmented out using “Color by Zone” (10 Å radius) and aligned to each other with “Fit in Map” in ChimeraX (version 1.71) (Fig. S5A).

Density for the individual bases were then compared visually and separated into three groups: density with an oval-like protrusion emanating directly from the sugar/phosphate backbone, bases containing a large density bending away from the backbone, and an ambiguous density not fitting the loop-like or bent profile. Comparison of the oval-like and bent density profiles to previously determined structures of bases suggested that these two groups belonged to purines and pyrimidines, respectively (Fig. S2). Bases belonging to each of these respective classes were labeled as either a purine (R) or pyrimidine (Y). Extending the analysis, we then compared bases within a single base pair to determine if each base pair contained both a purine and pyrimidine. In both the wrapped and not-wrapped structures, only two base pairs in each map did not contain both a purine and pyrimidine. Based on the structural assignment for each base, a simple, linear R/Y profile could then be constructed for each DNA strand.

Similarly, a sequence-based R/Y profile was constructed for the 601-bp minicircle DNA, whereby each Adenine and Guanine were assigned a “R”, and each Thymine and Cytosine were assigned a “Y”. The structure-based R/Y profiles were then compared to the forward and reverse directions of the minicircle DNA sequence profile. In the wrapped state, a single possible alignment where 22 of the 24 bases in the DNA pattern (R/Y) matched the sequence profile was identified at positions 112–135 in the minicircle DNA. Even allowing for the two mismatches, the probability of finding this pattern in “random” DNA is 1 in 55,738 (0.001794 %). Likewise, alignment of the 30-base profile from the not-wrapped structure revealed a unique sequence in the DNA minicircle profile, corresponding to positions 132 to 161 in the minicircle DNA, where 28 of the 30 base profiles matched (1 in 2,304,167 or 0.0000434 %).

For the wrapped structure, only 24 of the 30 possible base pairs in the G-segment DNA were used for the profile. The other base pairs were initially excluded from the profile as the density features of these bases was less well resolved from lower local resolution. However, after the initial alignment of the 24 bases in the structural profile, it was possible to extend the alignment to cover all 30 base pairs, corresponding to positions 109–138 of the minicircle DNA, at the G-segment.

To generate an atomistic model of the G-segment DNA in the wrapped and not-wrapped gyrase structures, the individual bases in the previously deposited structures (bases 84–118 in chain E of PDBID:8QDX and bases 1–30 in chain F of PDBID: 8QQS, respectively) were mutated to the corresponding nucleotides, which were extrapolated from the sequence/structure R/Y profile alignments. The resulting models were then iteratively refined nine times against the corresponding density maps with Coot and Phenix real_space_refine (nproc=4, run=minimization_global+local_grid_search+morphing+simulated_annealing+adp, resolution = 4). Computational and visual examination of the fit to density appeared to indicate this new assignment fit the density maps well. For the final models, chain E of the wrapped DNA-gyrase model corresponded to positions 104 through 138 in the minicircle DNA sequence. For the not-wrapped DNA G-segment-gyrase model, chain E corresponded to bases 132–161.

### Analysis of DNA sequence-dependent deformability

Our custom Python program utilized the sequence based deformability values of DNA base pair steps in a tetrameric context (grouped by four base pairs) to analyze the sequence-dependent deformability^21^ of the 601 bp minicircle. This program first applies periodic boundary conditions to the written sequence, considering the start site assigned in Vayssières *et al*. 2024^10^, copying the start onto the end and the end onto the start, allowing the sequence to be read as circular. Then a 4 bp sliding window rolls through the entire DNA sequence, identifying the tetramers and building a list of the sequence-dependent deformability values for every base pair step, according published base pair step parameters^20,21^.

To quantify local deformability along the minicircle, we implemented a *k*-mer-based sliding window analysis. For a given *k*-mer (a subsequence of length *k*), the deformability score was calculated as the average of all base pair step deformabilities within that segment. The chosen length, *k*, also defined the lengths of the sequences copied to establish periodic boundary conditions. This window was then slid one base at a time along the entire minicircle sequence, treating the circle with periodic boundary conditions to ensure continuity. In this way, we generated a deformability profile analogous to a hydrophobicity plot, allowing direct comparison of regional flexibility across the sequence. For this study, *k*-mers of length 35—matching the length of the G-segment—and length 49—matching the length of the β-pinwheel wrap—were chosen for analysis. The sliding window moves through the list of deformability values of the entire circular sequence, calculating the deformability score of each possible *k*-mer as it moves through them. Regions with highest deformability were then determined by comparing the averaged deformability scores of all *k*-mers on a sequence to each other.

### Analysis of gyrase-induced DNA deformation

The spatial arrangements of paired bases in gyrase-bound DNA structures were characterized, using the 3DNA/DSSR software^61,62^, in terms of the six rigid-body parameters that describe the precise orientation and displacement of the two bases. Three of the parameters are angles—so-called Buckle, Propeller, Opening^63^—describing the orientation of coordinate frames embedded in the base planes, and three are components of the displacement vector—so-called Shear, Stretch, Stagger^63^—joining the origins of the base frames^64^. We took advantage of the --pair-list option within the software to examine the features of base pairs expected based on the complementarity of chain sequence as well as the features of ‘new’ base pairs formed at sites of chain distortion. Base pairs with rigid-body parameters outside standard observed ranges^65^ were classified as ‘melted.’ The distortions of base-pair steps relative to those in canonical B-form DNA were similarly identified from extreme values of the six rigid-body step parameters relating coordinate frame on successive base pairs—the angles Tilt, Roll, Twist and the displacements Shift, Slide, Rise^63^. See^20,21^ for standard values.

The binding of proteins to DNA is known to induce conformational transitions of the double helix at individual base-pair steps^46,66^. Moreover, the DNA base-pair steps in such complexes may fall into different conformational categories, *i.e*., some steps may adopt under- or overwound conformations while others remain close to the canonical B-like form. We used the value of *z*_P_, the mean (out-of-plane) *z*-coordinates of the backbone phosphorus atoms with respect to the coordinate frame of a base-pair step^43^, to distinguish A-like from B-like steps. The classification scheme is based on previously established criteria^66^: B DNA *z*_P_ ≤ 0.5 Å; A DNA *z*_P_ ≥ 1.5 Å. The A-like steps are underwound relative to B DNA with lower Twist, positive Roll, and negative Slide. The characteristic pattern of overwound C-like DNA—higher Twist, negative Roll, and positive Slide^46^—does not occur in the DNA-gyrase complexes.

We also considered TA-DNA base-pair steps resembling the highly bent and untwisted steps found in the DNA complexed with the TATA-box-binding protein^67^. The TA form of the double helix is distinguished from B-DNA by z_P_(h), the projection, on the local helical axis, of the vector that links the phosphorus atoms on the two strands of a given base-pair step^61^. The dimer steps are classified here according to established guidelines^61^ B DNA z_P_(h) < 4 Å; TA-DNA z_P_(h) > 4 Å.

We related the sites of DNA deformation to the interactions with protein using the SNAP component of the 3DNA/DSSR software^68^. SNAP defines a local reference frame in the side chain of each amino acid and takes advantage of the standard base reference frame to determine the rigid-body parameters between closely spaced nucleotide and amino-acid residues. We counted the number of such contacts at each base pair and grouped the results in terms of protein chain and DNA strand identifiers.

### Comparison to the Topo-seq identified gyrase motif

The position weight matrix (PWM) of the gyrase motif was obtained from the supplementary information of the Topo-Seq paper^38,39^. Sequences of the negatively supercoiled 601 bp minicircle were then compared to the motif’s position weight matrix as described^38,39^ to obtain the motif score. To compare the 601 bp minicircle sequence to only parts of the motif, the PWM was split into three smaller position weight matrices—one containing the position corresponding to the first period region and the G-segment core of the motif, one containing only the positions corresponding to the G segment core, and one containing the positions corresponding to the G-segment core then the second period region of the motif. These 3 PWMs were then compared to the 601 bp minicircle to obtain motif scores as described^38,39^ (Table S5).

## Supplementary Material

**Fig. 3.**
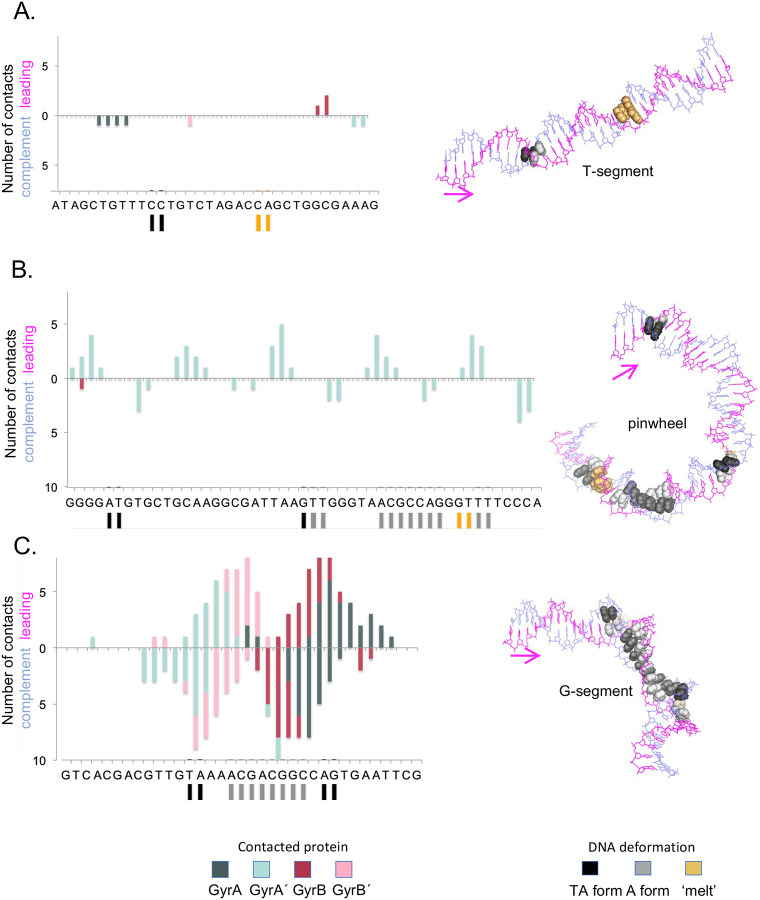
Distortions of DNA in the wrapped gyrase complex. The locations and numbers of amino-acid-nucleotide contacts, the sites of localized helical unwinding, and the identities of “melted” base pairs in the wrapped complex are reported as a function of chain sequence (left). Contacts are grouped by protein chain and DNA strand, with the terms GyrA, GyrB, GyrÁ, GyrB´, leading, and complement corresponding respectively to chains A–F in the updated pdb files. Sites of DNA deformation on the T-segment (A.), pinwheel (B.), and G-segment (C.) are highlighted by color-coded bars on the contact maps and by spheres on the associated molecular images. The leading DNA strand is depicted in magenta and the complementary strand in blue, with the chain direction denoted by the magenta arrow. The unwinding of DNA includes both A-like states and the highly distorted TA form originally found in complexes of DNA with the TATA-box binding protein^35,36^. See Fig. S6 for comparative contact plots and molecular images of the G-segments in the wrapped and not-wrapped complexes.

**Fig. 4.**
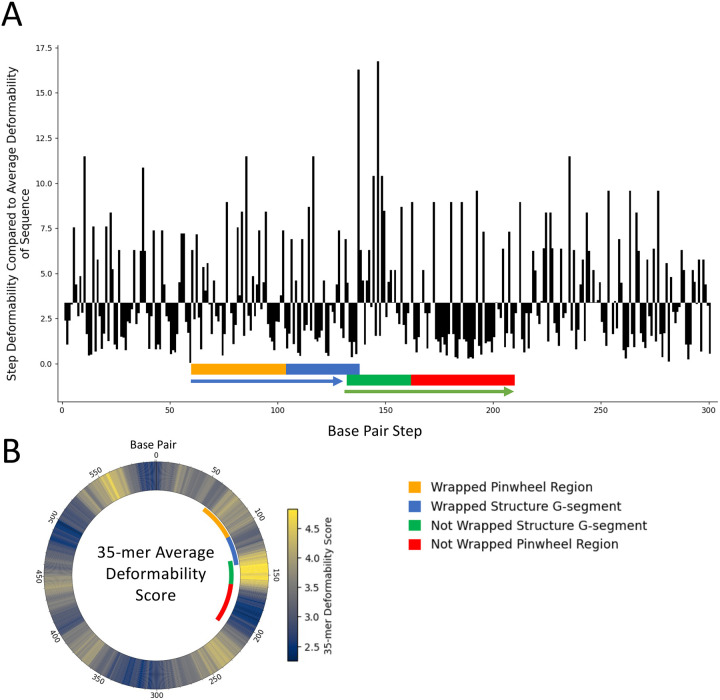
Minicircle DNA sequence-dependent deformability. A. The sequence-dependent deformability value of each pair step in the first 300 bp of the 601-bp minicircle DNA, centered on the overlap of the two gyrase G-segments (blue is wrapped and green is not-wrapped). The sequence of the DNA wrapped around the β-pinwheel is colored in orange and the sequence in the minicircle that did not wrap around the β-pinwheel is colored in red. The direction of the DNA relative to gyrase is indicated by the arrows (blue arrow is wrapped and green arrow is not-wrapped G-segments). The baseline of this bar plot was chosen to be 3.37 as this is the average deformability of all base pair steps in the 601 bp minicircle sequence. B. The sequence-dependent deformability score of a sliding *k*-mer (*k* = 35) along the entire 601-bp minicircle is shown. Each colored bp at the center of the 35-mer represents the deformability score of that bp and the +/− 17 bp surrounding it. The labels within the circular heatmap have the same color scheme as in (A). In yellow are the highest averaged *k*-mer deformability scores (~5.0) and the blue represents the lowest averaged *k*-mer deformability scores (~2.0). The circular heatmap was created with the Python library pycirclize^69^.

1 **Fig. S1**. **CryoREAD results**. Negatively supercoiled DNA minicircle G-segment models generated from CryoREAD for the wrapped (A) and not-wrapped (B) complexes are shown. The models superimposed on the density (right) fail to fit the density map. The coloring scheme is the same as in Fig. 2.
**Fig. S2. Structural superimposability of the G-segments**. The wrapped (A) and not-wrapped (B) G-segment models are shown superimposed on their respective G-segment density. In (C), the models are superimposed with the colored bases representing the palindrome.
**Fig. S3. Using k-means analysis to describe DNA sequence-dependent deformability**. The *k*-means approach is similar to the approach of Kyte and Doolittle^44^ for hydropathy analysis for protein sequences (see text). (A) Schematic for the sliding window approach. The deformability value, V_step_, in units of deg^3^Å^3^ from each tetrameric DNA sequence (in brackets and shown in Fig. 4) is averaged for a given length of DNA (*e.g*., the length of the G-segment) along a sliding window one base at a time. (B) Deformability scores for *k* = 35 (the length of the bound G-segment for the wrapped gyrase-minicircle structure) are shown as a scatter plot akin to classic protein hydropathy plots^44^. The *y*-axis shows the difference between the 35-mer deformability score and the average deformability of all base pair steps within the 601 bp minicircle sequence. The *x*-axis shows the base pair numbers at the center of each 35-mer, according to the numeric of Vayssières et al. 2024^10^. Circular heatmap of the sequence-dependent deformability scores of a sliding *k*-mer window (C) (*k* = 35) (D) (*k* = 49) or along the entire 601 bp minicircle. Each colored base pair on the heatmap represents the deformability score of the *k*-mer centered on each base pair and +/− 17 bp (C) or ± 24 bp (D) around it. The DNA sequence wrapped around the β-pinwheel is labeled orange, the G-segment of the wrapped structure is labeled blue, the G-segment of the not wrapped structure is labeled green, and the sequence of the minicircle that did not wrap around the β-pinwheel is labeled red.
**Fig. S4. Comparative influence of localized base-pair step deformation on right-handed (negative) and left-handed (positive) global DNA folding**. Molecular representations of chains of 55 base pairs with 5-bp stretches of A-like or C-like DNA interspersed between 5-bp stretches of ideal B-DNA. Models constructed using the ‘Customized base-pair-step/nucleotide parameters’ rebuilding module of Web 3DNA 2.0^22^. Base pairs assumed to be planar in ideal Watson-Crick arrangements^64^. Rigid-body parameters describing base-pair steps assigned the following Shift, Slide, Rise, Tilt, Roll, Twist values for B DNA: 0 Å, 0 Å, 3.4 Å, 0°, 0°, 36°; A DNA: 0 Å, −2.0 Å, 3.4 Å, 0°, 12°, 34°; C DNA: 0 Å, 2.0 Å, 3.4 Å, 0°, −12°, 38.298°. Note (i) the identical placement of both the initial base (red lines) and the chain direction (magenta arrow), (ii) the different modes of localized deformation, and (iii) the distinctly different pathways of the two superhelical models.
**Fig. S5**. **Comparison of existing G-segment structures**. Shown in (A) is a published structure of gyrase bound to linear MuSGS DNA wrapped around both β-pinwheels of gyrase^41^. A zoomed in view of the G-segment DNA, colored as in Fig. 2, is shown. In (B) and (C), a portion of the G-segment DNA is shown for the negatively supercoiled minicircle DNA^10^ and linear MuSGS^41^ DNA structures, respectively. A conserved RYRYYYYR pattern is located next to the G-segment bend and in a stretch of DNA that adopts a more open, A-form DNA structure (boxed region).
**Fig. S6. Comparative contact plots and molecular images of the gyrase-induced distortions of the G-segments of DNA in the wrapped and not-wrapped complexes**. Locations and numbers of amino-acid-nucleotide contacts are grouped by protein chain and DNA strand. Sites of DNA deformation are highlighted by color-coded bars on the contact maps and spheres on the molecular images. See the legend to Fig. 3 for further details.

**Supplementary information** is available for this paper at https://doi.org/…

This is a list of supplementary files associated with this preprint. Click to download.

- NCOMMS256005softwareinfo.zip

## Figures and Tables

**Table 1.** Purine/pyrimidine assignment derived from the density map for the well-resolved region of the DNA G-segment in the gyrase map with DNA wrapped around the β-pinwheel.

Reference Position (Chain E)	Base Assignment (R/Y)	Minicircle Pattern (R/Y)		Base Assignment (R/Y)	Reference Position (Chain F)
92	R	R	Y	Y	27
93	Y	Y	R	R	26
94	Y	Y	R	R	25
95	R	R	Y	Y	24
96	Y	Y	R	R	23
97	R	R	Y	Y	22
98	R	R	Y	Y	21
99	R	R	Y	Y	20
100	R	R	Y	Y	19
101	Y	Y	R	R	18
102	Y	R	Y	R	17
103	R	R	Y	Y	16
104	Y	Y	R	R	15
105	R	R	Y	Y	14
106	R	R	Y	Y	13
107	Y	Y	R	R	12
108	Y	Y	R	R	11
109	R	R	Y	Y	10
110	X	R	Y	X	9
111	Y	Y	R	R	8
112	R	R	Y	Y	7
113	R	R	Y	Y	6
114	R	R	Y	Y	5
115	Y	Y	R	R	4

Disagreement between the base hunting method and sequence is highlighted red; where the density was unresolvable is highlighted yellow.

**Table 2.** Purine/pyrimidine assignment derived from the density map for the well-resolved region of the DNA G-segment in the gyrase map without the β-pinwheel.

Reference Position (Chain E)	Base Assignment (R/Y)	Minicircle Pattern (R/Y)		Base Assignment (R/Y)	Reference Position (Chain F)
1	R	R	Y	Y	30
2	R	R	Y	Y	29
3	R	R	Y	Y	28
4	Y	Y	R	R	27
5	Y	Y	R	R	26
6	Y	Y	R	R	25
7	R	R	Y	Y	24
8	R	R	Y	Y	23
9	R	R	Y	Y	22
10	Y	Y	R	R	21
11	Y	Y	R	R	20
12	Y	Y	R	R	19
13	R	R	Y	Y	18
14	R	R	Y	Y	17
15	Y	Y	R	R	16
16	R	R	Y	Y	15
17	Y	Y	R	R	14
18	Y	Y	R	R	13
19	R	R	Y	Y	12
20	R	R	Y	Y	11
21	R	R	Y	Y	10
22	R	R	Y	Y	9
23	R	R	Y	Y	8
24	R	R	Y	Y	7
25	R	R	Y	Y	6
26	R	Y	R	Y	5
27	R	R	Y	Y	4
28	R	R	Y	Y	3
29	Y	Y	R	R	2
30	R	Y	R	Y	1

Disagreements between the base hunting method and sequence are highlighted red

## Data Availability

All data supporting the findings of this study are available in the article and Supplementary Information files. Source data are provided in this paper.
