# Supplementary material for: DNA deformability defines sequence-dependent capture of E. coli gyrase
Source: Res Sq. 2025 Aug 18:rs.3.rs-7265879. Preprint. [Version 1] doi: 10.21203/rs.3.rs-7265879/v1 (PMC12393610; doi:10.21203/rs.3.rs-7265879/v1)
Supplement: 1 — Fig. S1. CryoREAD results. Negatively supercoiled DNA minicircle G-segment models generated from CryoREAD for the wrapped (A) and not-wrapped (B) complexes are shown. The models superimposed on the density (right) fail to fit the density map. The coloring scheme is the same as in Fig. 2. Fig. S2. Structural superimposability of the G-segments. The wrapped (A) and not-wrapped (B) G-segment models are shown superimposed on their respective G-segment density. In (C), the models are superimposed with the colored bases representing the palindrome. Fig. S3. Using k-means analysis to describe DNA sequence-dependent deformability. The k-means approach is similar to the approach of Kyte and Doolittle44 for hydropathy analysis for protein sequences (see text). (A) Schematic for the sliding window approach. The deformability value, Vstep, in units of deg3Å3 from each tetrameric DNA sequence (in brackets and shown in Fig. 4) is averaged for a given length of DNA (e.g., the length of the G-segment) along a sliding window one base at a time. (B) Deformability scores for k = 35 (the length of the bound G-segment for the wrapped gyrase-minicircle structure) are shown as a scatter plot akin to classic protein hydropathy plots44. The y-axis shows the difference between the 35-mer deformability score and the average deformability of all base pair steps within the 601 bp minicircle sequence. The x-axis shows the base pair numbers at the center of each 35-mer, according to the numeric of Vayssières et al. 202410. Circular heatmap of the sequence-dependent deformability scores of a sliding k-mer window (C) (k = 35) (D) (k = 49) or along the entire 601 bp minicircle. Each colored base pair on the heatmap represents the deformability score of the k-mer centered on each base pair and +/− 17 bp (C) or ± 24 bp (D) around it. The DNA sequence wrapped around the β-pinwheel is labeled orange, the G-segment of the wrapped structure is labeled blue, the G-segment of the not wrapped structure is la [file NIHPPRS7265879V1-supplement-1.pdf]

Tables 3–6: See Excel File

A.

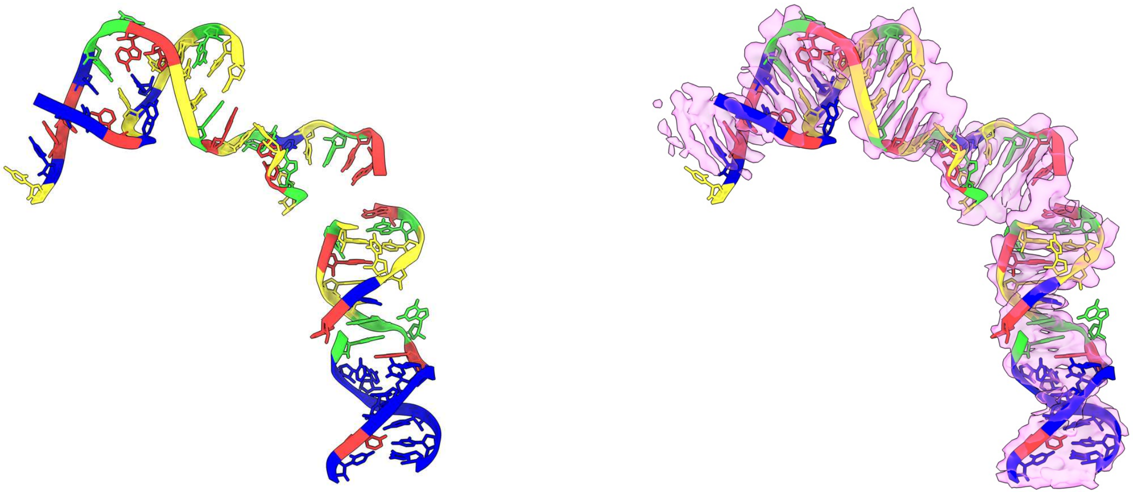

B.

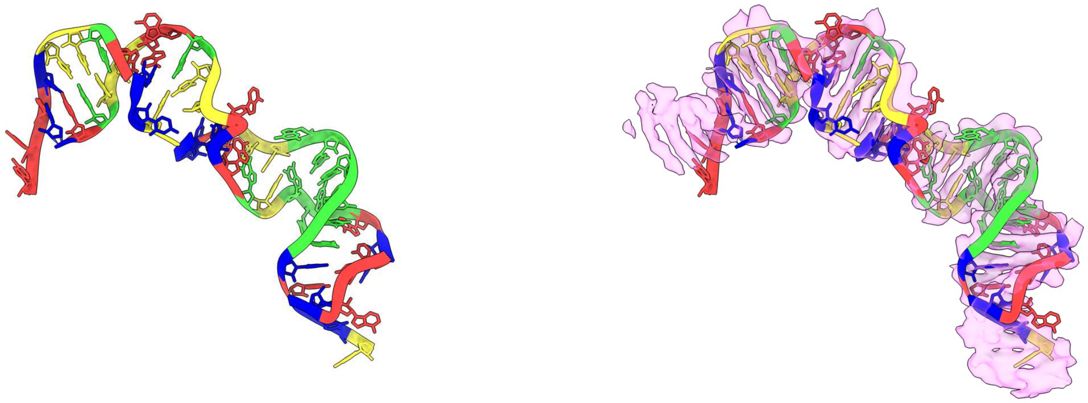

Figure S1

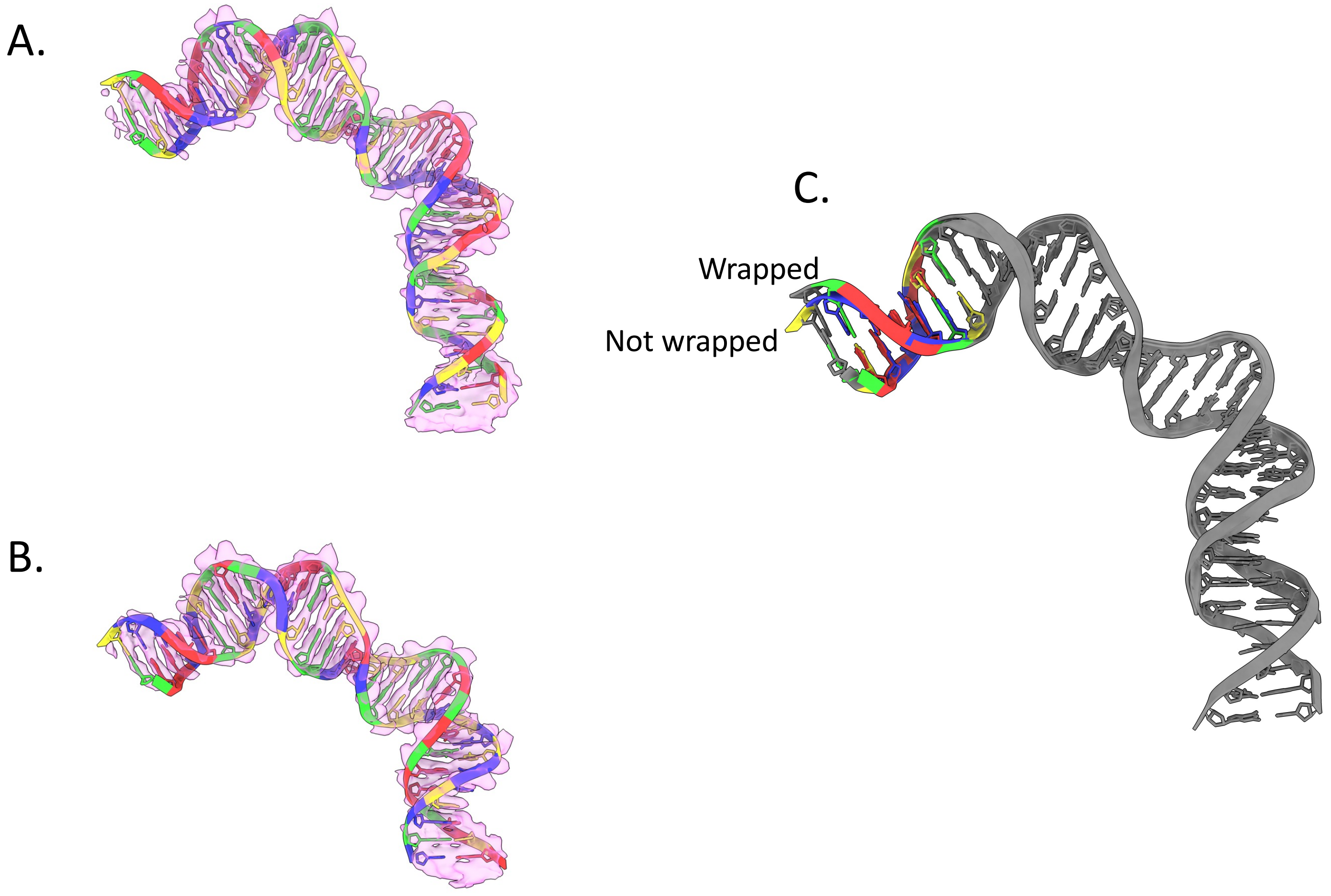

Figure S2

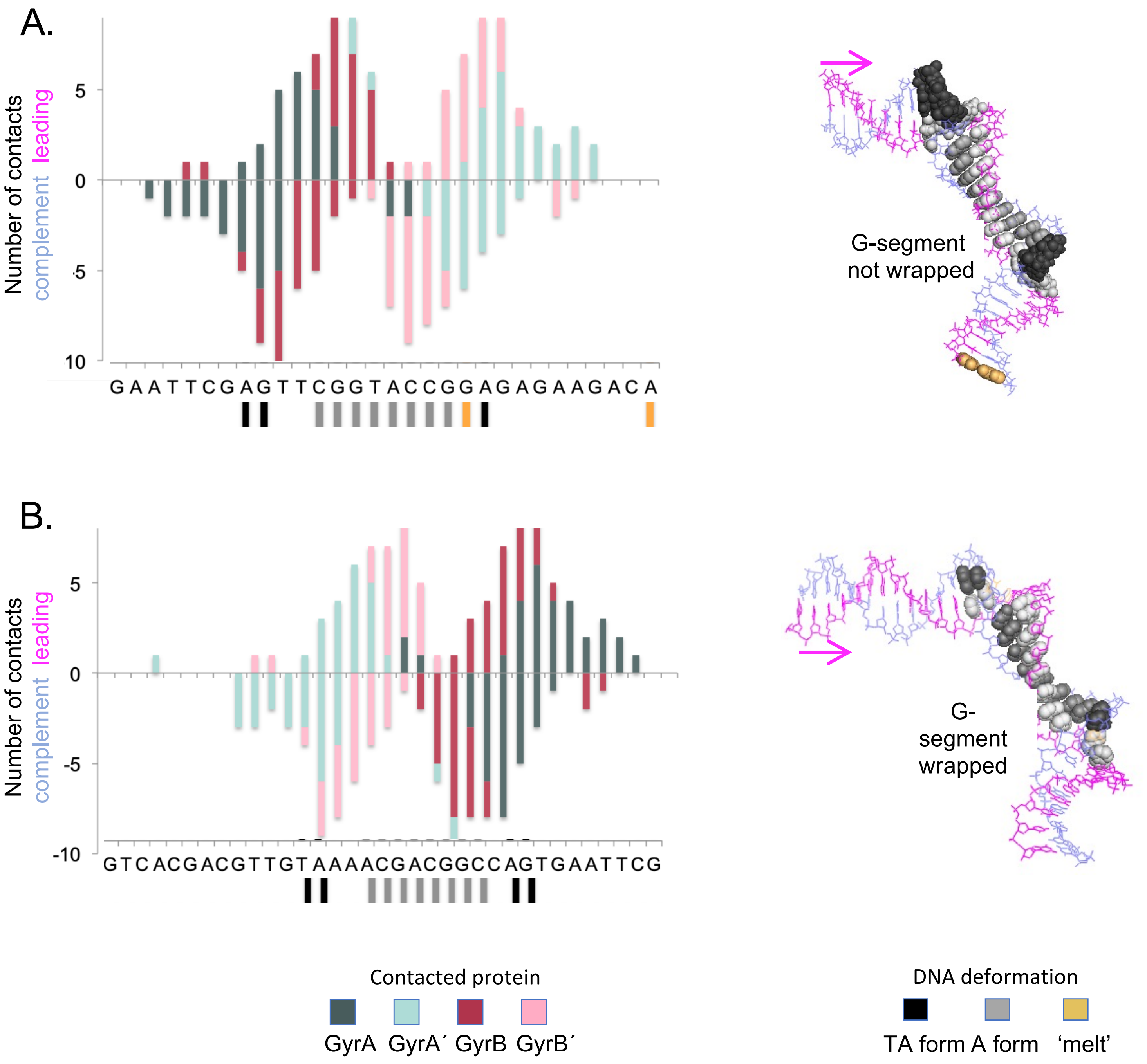
A.
Number of contacts
complement leading
5
0
-5
-10
G A A T T C G A G T T C G G T A C C G G A G A G A A G A C A
G-segment not wrapped
B.
Number of contacts
complement leading
5
0
-5
-10
G T C A C G A C G T T G T A A A A C G A C G G C C A G T G A A T T C G
G-segment wrapped
Contacted protein
GyrA GyrA´ GyrB GyrB´
DNA deformation
TA form A form 'melt'

Figure S3

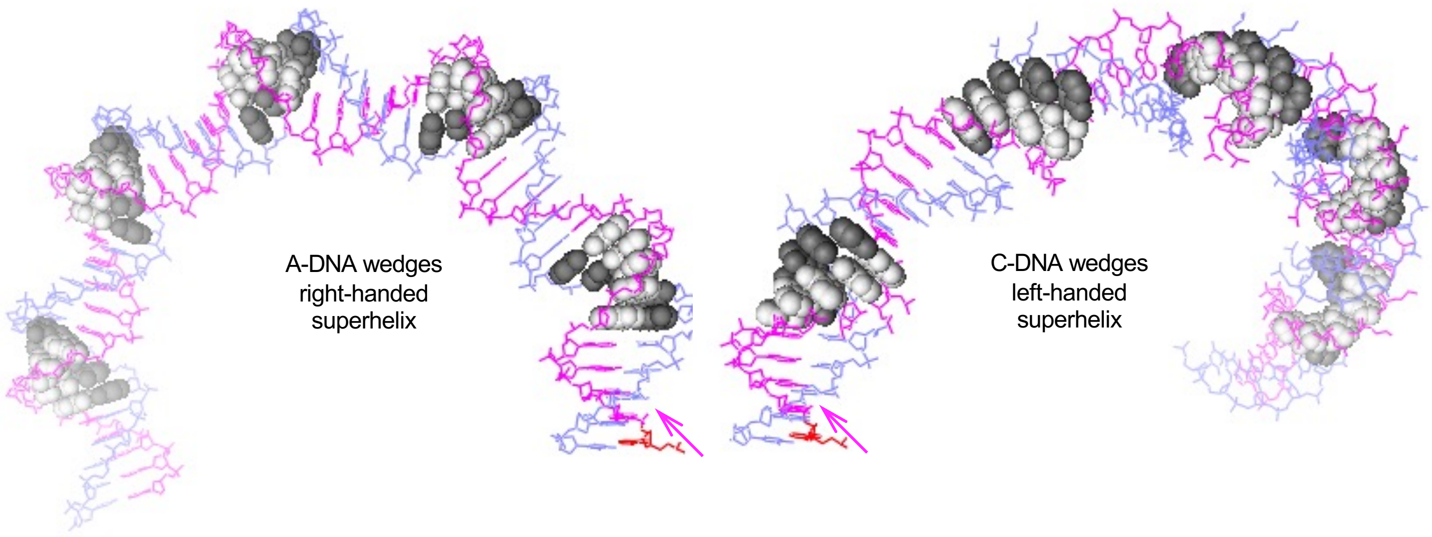
A-DNA wedges
right-handed
superhelix
C-DNA wedges
left-handed
superhelix

Figure S4

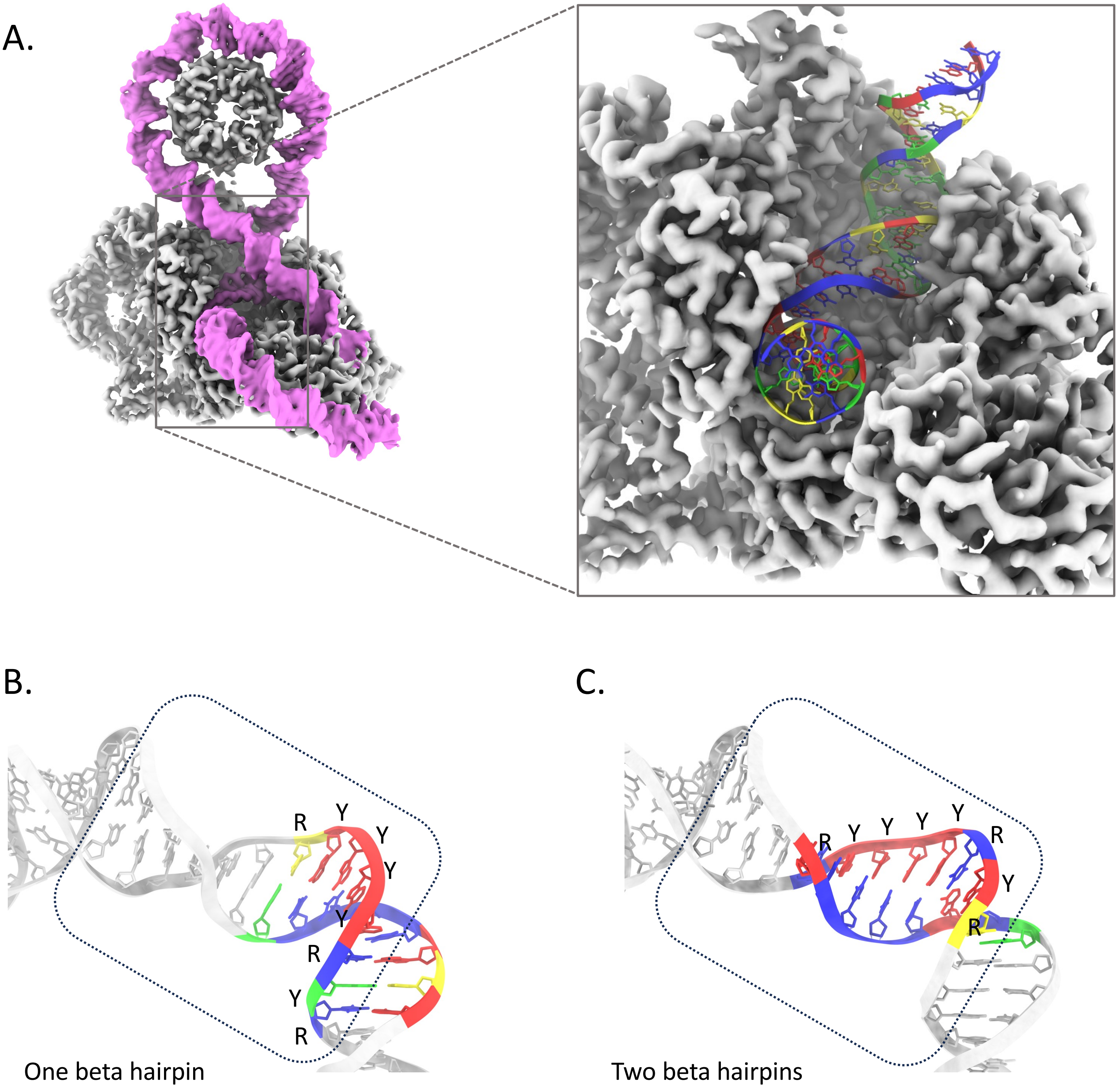

Figure S5

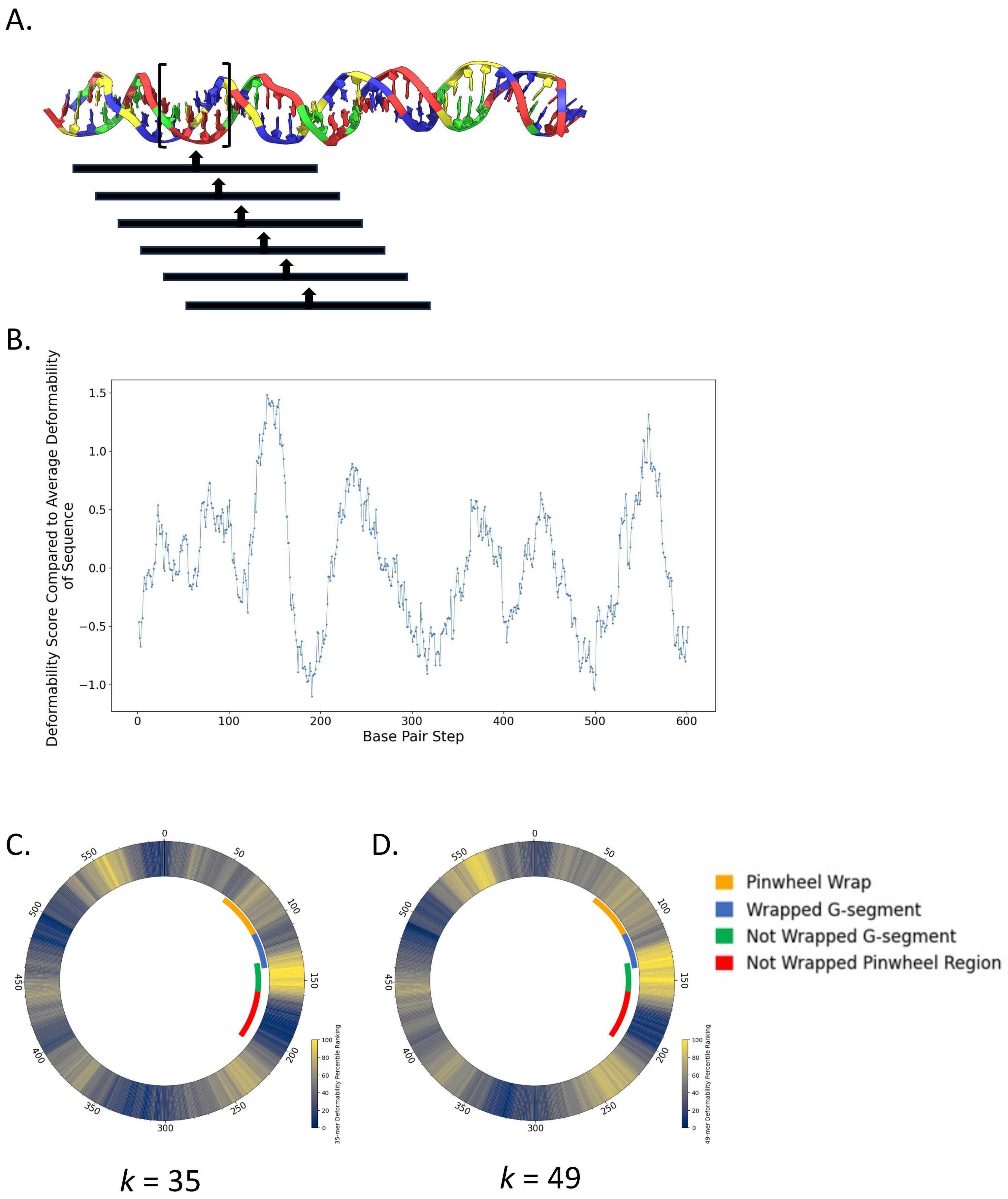
A.
B.
Deformability Score Compared to Average Deformability of Sequence
Base Pair Step
1.5
1.0
0.5
0.0
−0.5
−1.0
0
100
200
300
400
500
600
C.
D.
Pinwheel Wrap
Wrapped G-segment
Not Wrapped G-segment
Not Wrapped Pinwheel Region
35-mer Deformability Percentile Ranking
49-mer Deformability Percentile Ranking
k = 35
k = 49

Figure S6

Table S1. Model Statistics*

	Wrapped	Not wrapped
Initial model used (PDB)	8QDX	8QQS
Model resolution (Å)	3.0	3.1
FSC threshold	0.143	0.143
Map Model CC (mask)	0.81	0.86
**Model composition**		
Chains	6	6
Non-hydrogen atoms	27,407	21,197
Protein residues	2,867	2,524
Nucleotides	236	60
***B* factors ($Å^2$)**		
Protein	132.69	82.49
Nucleotide	234.87	73.97
**R.M.S. deviations**		
Bond lengths (Å)	0.003	0.003
Bond angles (°)	0.576	0.504
**Validation**		
MolProbity score	1.38	1.23
Clashscore	4.15	2.45
Poor rotamers (%)	1.20	1.13
**Ramachandran plot**		
Favored (%)	97.37	97.05
Allowed (%)	2.63	2.95
Disallowed (%)	0	0

*Model quality metrics and agreement with the density map were calculated with Phenix for both the updated/refined wrapped and not wrapped maps and models.

Center Base	Sequence	Deformability Score
1	TCGTTTTTG	2.957296
2	CGTTTTTGC	2.842753
3	GTTTTTGCA	2.877891
4	TTTTTGCAT	2.870453
5	TTTTGCATC	2.870453
6	TTTGCATCA	2.860183
7	TTGCATCAG	2.883089
8	TGCATCAG	2.906136
9	GCATCAGG	2.852752
10	CATCAGGA	2.899206
11	ATCAGGAA	2.933042
12	TCAGGAAA	2.990508
13	CAGGAAAG	3.110045
14	AGGAAAGC	3.311837
15	GGAAAGCA	3.379407
16	GAAAGCAG	3.39115
17	AAAGCAGA	3.359412
18	AAGCAGAA	3.39863
19	AGCAGAAG	3.538995
20	GCAGAAGC	3.506219
21	CAGAAGCT	3.474787
22	AGAAGCTTG	3.380087
23	GAAGCTTG	3.485357
24	AAGCTTGG	3.541926
25	AGCTTGGC	3.582047
26	GCTTGGCG	3.581088
27	CTTGGCGT	3.570302
28	TTGGCGTA	3.536248
29	TGGCGTAA	3.480907
30	GGCGTAAT	3.358088
31	GCGTAATC	3.360452
32	CGTAATCAT	3.456031
33	GTAATCATG	3.505108
34	TAATCATGG	3.494279
35	AATCATGGT	3.290795
36	ATCATGGTC	3.257767
37	TCATGGTCA	3.37941
38	CATGGTCA	3.419911
39	ATGGTCATA	3.411151
40	TGGTCATAG	3.45031
41	GGTCATAG	3.347157
42	GTCATAGC	3.403917
43	TCATAGCTG	3.471537
44	CATAGCTG	3.553134
45	ATAGCTGTT	3.463455
46	TAGCTGTTT	3.471625
47	AGCTGTTTC	3.392969

48	GCTGTTTC	3.338288
49	CTGTTTCC	3.364975
50	TGTTTCCT	3.414932
51	GTTTCCTG	3.293188
52	TTTCCTGT	3.296348
53	TTCCTGTC	3.452921
54	TCCTGTCT	3.507792
55	CCTGTCTA	3.517321
56	CTGTCTAG	3.492968
57	TGTCTAGA	3.475048
58	GTCTAGAC	3.501199
59	TCTAGACC	3.534356
60	CTAGACCA	3.650974
61	TAGACCAG	3.55311
62	AGACCAG	3.566259
63	GACCAGC	3.491449
64	ACCAGCTG	3.47129
65	CCAGCTG	3.555433
66	CAGCTGG	3.555295
67	AGCTGGC	3.493377
68	GCTGGCG	3.630105
69	CTGGCGA	3.67079
70	TGGCGAAA	3.747964
71	GGCGAAA	3.769569
72	GCGAAAG	3.723802
73	CGAAAGG	3.699125
74	GAAAGGG	3.6763
75	AAAGGGG	3.680678
76	AAGGGGG	3.713855
77	AGGGGGA	3.74872
78	GGGGGAT	3.793044
79	GGGGATG	3.852598
80	GGGATGTG	3.74287
81	GGATGTGC	3.610009
82	GATGTGCT	3.596381
83	ATGTGCTG	3.704449
84	TGTGCTGC	3.725964
85	GTGCTGCA	3.690927
86	TGCTGCAA	3.652551
87	GCTGCAA	3.512151
88	CTGCAAG	3.602481
89	TGCAAGG	3.624038
90	GCAAGGC	3.557232
91	CAAGGCG	3.653775
92	AAGGCGAT	3.575934
93	AGGCGATT	3.746736
94	GGCGATTA	3.740242
95	GCGATTAA	3.6701

96	CGATTAAG	3.645424
97	GATTAAGT	3.635164
98	ATTAAGTT	3.664302
99	TTAAGTTG	3.667783
100	TAAGTTGG	3.642727
101	AAGTTGGG	3.523753
102	AGTTGGGT	3.513299
103	GTTGGGTA	3.50172
104	TTGGGTAA	3.57091
105	TGGGTAAC	3.679746
106	GGGTAACG	3.563013
107	GGTAACGC	3.514008
108	GTAACGCC	3.482766
109	TAACGCCA	3.5438
110	AACGCCAG	3.329545
111	ACGCCAGG	3.281897
112	CGCCAGGG	3.268592
113	GCCAGGGT	3.178632
114	CCAGGGTT	3.463419
115	CAGGGTTT	3.503152
116	AGGGTTTT	3.445652
117	GGGTTTTC	3.404967
118	GGTTTTCC	3.40702
119	GTTTTCCC	3.36323
120	TTTTCCCA	3.382458
121	TTTCCCAG	3.569145
122	TTCCCAGT	3.575673
123	TCCCAGTC	3.90904
124	CCCAGTCA	3.892661
125	CCAGTCAC	4.061245
126	CAGTCACG	4.159171
127	AGTCACGA	4.059594
128	GTCACGAC	4.113477
129	TCACGACG	4.204035
130	CACGACGT	4.240647
131	ACGACGTT	4.204633
132	CGACGTTG	4.240584
133	GACGTTGT	4.19022
134	ACGTTGTA	4.358573
135	CGTTGTAA	4.394244
136	GTTGTAAA	4.272973
137	TTGTAAAA	4.292843
138	TGTAAAAC	4.318542
139	GTAAAACG	4.323917
140	TAAAACGA	4.313633
141	AAAACGAC	4.08749
142	AAACGACG	4.090018
143	AACGACGG	4.13524

144	ACGACGG(	4.213191
145	CGACGGC(	4.233351
146	GACGGCC/	4.154774
147	ACGGCCA(	4.20023
148	CGGCCAG	4.2616
149	GGCCAGT(	4.380574
150	GCCAGTGA	4.348506
151	CCAGTGAA	4.32765
152	CAGTGAAT	4.249204
153	AGTGAATT(	4.126074
154	GTGAATTC(	4.107907
155	TGAATTCG/	4.087512
156	GAATTCGA(	3.977699
157	AATTCGAG(	4.070844
158	ATTCGAGC	4.07987
159	TTCGAGCT(	4.07987
160	TCGAGCTC	4.060725
161	CGAGCTC(	4.077453
162	GAGCTCG(	3.924563
163	AGCTCGGT	3.82109
164	GCTCGGTA	3.750854
165	CTCGGTAC	3.734833
166	TCGGTACC	3.64653
167	CGGTACC(	3.521094
168	GGTACCG(	3.483894
169	GTACCGGA	3.466924
170	TACCGGA(	3.455056
171	ACCGGAG/	3.116812
172	CCGGAGA(	3.236767
173	CGGAGAG/	3.043881
174	GGAGAGA(	2.893057
175	GAGAGACA	2.864897
176	AGAGACAA	2.783726
177	GAGACAAC	2.706248
178	AGACAACT	2.706248
179	GACAACTT.	2.661283
180	ACAACTTA/	2.655371
181	CAACTTAA/	2.742524
182	AACTTAAA(	2.580599
183	ACTTAAAG/	2.571188
184	CTTAAAGA(	2.701042
185	TTAAAGAG/	2.677217
186	TAAAGAGA(	2.624143
187	AAAGAGAC	2.495871
188	AAGAGACT	2.538393
189	AGAGACTT.	2.711727
190	GAGACTTA.	2.709199
191	AGACTTAA/	2.651349

192	GACTTAAA/	2.601699
193	ACTTAAAA(	2.560501
194	CTTAAAAG/	2.601699
195	TTAAAAGAT	2.673655
196	TAAAAGATT	2.710828
197	AAAAGATT/	2.579703
198	AAAGATTA/	2.597675
199	AAGATTAAT	2.644411
200	AGATTAATT	2.764425
201	GATTAATTT	2.908615
202	ATTAATTTA,	2.912025
203	TTAATTTAA,	3.077324
204	TAATTTAAA	3.176425
205	AATTTAAAA	3.036699
206	ATTTAAAAT	3.038444
207	TTTAAAATT	3.05117
208	TTAAAATTT,	3.177948
209	TAAAATTTA	3.202682
210	AAAATTTAT	3.07441
211	AAATTTATC	3.116932
212	AATTTATCA	3.330447
213	ATTTATCAA	3.362695
214	TTTATCAAA	3.486005
215	TTATCAAA/	3.496897
216	TATCAAAA/	3.508322
217	ATCAAAAA(	3.400289
218	TCAAAAAG,	3.499791
219	CAAAAAGA	3.515342
220	AAAAAGAG	3.537657
221	AAAAGAGT,	3.644113
222	AAAGAGTA	3.689335
223	AAGAGTATT	3.77142
224	AGAGTATT(	3.82927
225	GAGTATTG/	3.872395
226	AGTATTGA(	3.895356
227	GTATTGACT	3.880474
228	TATTGACTT	3.863693
229	ATTGACTT/	3.751458
230	TTGACTTA/	3.931903
231	TGACTTAA/	3.967013
232	GACTTAAA(	3.849104
233	ACTTAAAGT	3.88723
234	CTTAAAGT(	3.910748
235	TTAAAGTCT	3.996412
236	TAAAGTCT/	4.019373
237	AAAGTCTA/	3.848396
238	AAGTCTAA(	3.826432
239	AGTCTAAC(	3.832466

240	GTCTAACC	3.973883
241	TCTAACCT	3.990642
242	CTAACCTA	3.951324
243	TAACCTAT	3.995731
244	AACCTATA	4.018347
245	ACCTATAG	3.998016
246	CCTATAGG	3.977225
247	CTATAGGA	3.922694
248	TATAGGAT	3.909468
249	ATAGGATA	3.789618
250	TAGGATAC	3.796243
251	AGGATACT	3.754339
252	GGATACTT	3.646854
253	GATACTTA	3.799724
254	ATACTTAC	3.797979
255	TACTTACA	3.785253
256	ACTTACAG	3.687851
257	CTTACAGC	3.751243
258	TTACAGCC	3.695677
259	TACAGCCA	3.725285
260	ACAGCCAT	3.545916
261	CAGCCATA	3.554409
262	AGCCATAG	3.483313
263	GCCATAGA	3.531584
264	CCATAGAG	3.623632
265	CATAGAGA	3.639899
266	ATAGAGAG	3.542231
267	TAGAGAGG	3.52127
268	AGAGAGGG	3.368966
269	GAGAGGGA	3.34666
270	AGAGGGAT	3.369981
271	GAGGGATA	3.331081
272	AGGGATAA	3.380019
273	GGGATAAG	3.320698
274	GGATAAGG	3.346675
275	GATAAGGT	3.390256
276	ATAAGGTG	3.421092
277	TAAGGTGA	3.412338
278	AAGGTGAA	3.213745
279	AGGTGAAA	3.153997
280	GGTGAAAT	3.271906
281	GTGAAATA	3.223105
282	TGAAATAA	3.188176
283	GAAATAAT	3.063483
284	AAATAATA	3.169773
285	AATAATAG	3.182123
286	ATAATAGA	3.202007
287	TAATAGAA	3.212734

288	AATAGAAT(	3.051448
289	ATAGAATG(	3.113266
290	TAGAATGG'	3.10521
291	AGAATGGT,	2.946142
292	GAATGGTA'	2.829124
293	AATGGTAT/	2.828448
294	ATGGTATA/	2.899329
295	TGGTATAAT	2.920628
296	GGTATAATT	2.896704
297	GTATAATTG	2.93155
298	TATAATTGC	2.923597
299	ATAATTGC(	2.831364
300	TAATTGCG(	2.822988
301	AATTGCGG	2.636116
302	ATTGCGGC	2.941427
303	TTGCGGCC	2.944845
304	TGCGGCC(	2.925084
305	GCGGCCG,	2.822251
306	CGGCCGA(	2.84999
307	GGCCGAG/	2.808505
308	GCCGAGAT	2.765885
309	CCGAGATC	2.75969
310	CGAGATCT	2.85328
311	GAGATCTC	2.866898
312	AGATCTCC	2.831166
313	GATCTCCA	2.769937
314	ATCTCCAT(	2.843585
315	TCTCCATG(	2.872715
316	CTCCATGG	2.863595
317	TCCATGGC	2.771239
318	CCATGGCA	2.688047
319	CATGGCAT	2.714967
320	ATGGCATC	2.633371
321	TGGCATCA	2.649094
322	GGCATCAA	2.554933
323	GCATCAAA	2.491194
324	CATCAAAT/	2.624178
325	ATCAAATA/	2.632932
326	TCAAATAA/	2.654167
327	CAAATAAA/	2.691781
328	AAATAAAA(	2.670541
329	AATAAAAC(	2.677733
330	ATAAAACG,	2.716519
331	TAAAACGA,	2.878444
332	AAAACGAA	2.70972
333	AAACGAAA	2.711774
334	AACGAAAG	2.709709
335	ACGAAAGC	2.860907

336	CGAAAGG(	2.868083
337	GAAAGGCT	2.784957
338	AAAGGCTC	2.838886
339	AAGGCTCA	3.039977
340	AGGCTCAG	3.067649
341	GGCTCAGT	3.177174
342	GCTCAGTC	3.174594
343	CTCAGTCG	3.148026
344	TCAGTCGA	3.090355
345	CAGTCGAA	3.101872
346	AGTCGAAA	3.099727
347	GTCGAAAG	3.155303
348	TCGAAAGA	3.208997
349	CGAAAGAC	3.304104
350	GAAAGACT	3.05905
351	AAAGACTG	3.09468
352	AAGACTGG	3.141772
353	AGACTGGG	3.152974
354	GACTGGG(	3.21216
355	ACTGGGC(	3.216555
356	CTGGGCCT	3.251841
357	TGGGCCTT	3.547663
358	GGGCCTTT	3.527313
359	GGCCTTTC	3.495396
360	GCCTTTCG	3.412027
361	CCTTTCGT	3.410912
362	CTTTCGTTT	3.496102
363	TTTCGTTTT	3.485463
364	TTCGTTTTA	3.478206
365	TCGTTTTAT	3.471975
366	CGTTTTATC	3.496655
367	GTTTTATCT	3.454711
368	TTTTATCTG	3.516355
369	TTTATCTGT	3.541032
370	TTATCTGTT	3.575179
371	TATCTGTTG	3.723924
372	ATCTGTTGT	3.612138
373	TCTGTTGTT	3.639717
374	CTGTTGTTT	3.711767
375	TGTTGTTTG	3.759535
376	GTTGTTTGT	3.650722
377	TTGTTTGTC	3.719804
378	TGTTTGTC(	3.729063
379	GTTTGTCG(	3.583762
380	TTTGTCGGT	3.562134
381	TTGTCGGT(	3.538116
382	TGTCGGTG	3.538116
383	GTCGGTGA	3.509267

384	TCGGTGAA	3.557088
385	CGGTGAAC	3.557044
386	GGTGAACG	3.53033
387	GTGAACGC	3.457409
388	TGAACGCT	3.45493
389	GAACGCTC	3.355732
390	AACGCTCT	3.378376
391	ACGCTCTC	3.368309
392	CGCTCTCC	3.378569
393	GCTCTCCT	3.288609
394	CTCTCCTG	3.280869
395	TCTCCTGA	3.234417
396	CTCCTGAG	3.216765
397	TCCTGAGT	3.224885
398	CCTGAGTA	3.175244
399	CTGAGTAG	3.166828
400	TGAGTAGG	3.171738
401	GAGTAGGA	3.207311
402	AGTAGGAC	3.164374
403	GTAGGACA	3.197073
404	TAGGACAA	3.232326
405	AGGACAAA	3.072667
406	GGACAAAT	2.993441
407	GACAAATC	3.041002
408	ACAAATCC	3.102285
409	CAAATCCG	3.150728
410	AAATCCGC	3.144738
411	AATCCGCC	3.161847
412	ATCCGCCG	3.243743
413	TCCGCCGG	3.326063
414	CCGCCGG	3.344842
415	CGCCGGGA	3.390891
416	GCCGGGA	3.378747
417	CCGGGAG	3.419757
418	CGGGAGC	3.426477
419	GGGAGCG	3.36136
420	GGAGCGG	3.336116
421	GAGCGGAT	3.33486
422	AGCGGATT	3.252767
423	GCGGATTT	3.165762
424	CGGATTTG	3.244644
425	GGATTTGA	3.199245
426	GATTTGAA	3.240904
427	ATTTGAAC	3.292938
428	TTTGAACG	3.383649
429	TTGAACGT	3.432343
430	TGAACGTT	3.500227
431	GAACGTTG	3.467905

432	AACGTTGC	3.400748
433	ACGTTGCG	3.587664
434	CGTTGCGA	3.577178
435	GTTGCGAA	3.463412
436	TTGCGAAG	3.447476
437	TGCGAAGC	3.443235
438	GCGAAGC	3.438737
439	CGAAGCA	3.502736
440	GAAGCAAC	3.54573
441	AAGCAAC	3.688116
442	AGCAACG	3.736248
443	GCAACGG	3.782606
444	CAACGGC	3.795903
445	AACGGCC	3.691602
446	ACGGCCC	3.647959
447	CGGCCCG	3.620247
448	GGCCCGG	3.704997
449	GCCCGGA	3.668785
450	CCCGGAG	3.628045
451	CCGGAGG	3.540158
452	CGGAGGG	3.486379
453	GGAGGGT	3.496261
454	GAGGGTG	3.512703
455	AGGGTGG	3.467943
456	GGGTGGC	3.44778
457	GGTGGCG	3.465305
458	GTGGCGG	3.338431
459	TGGCGGG	3.332636
460	GGCGGGC	3.259314
461	GCGGGCA	3.202069
462	CGGGCAG	3.243401
463	GGGCAGG	3.190047
464	GGCAGGA	3.193801
465	GCAGGAC	3.218142
466	CAGGACG	3.152533
467	AGGACGC	3.076856
468	GGACGCC	3.061016
469	GACGCCC	3.076856
470	ACGCCCG	3.08898
471	CGCCCGC	3.114359
472	GCCCGCC	3.022279
473	CCCGCCA	3.035201
474	CCGCCAT	2.993542
475	CGCCATAA	2.941508
476	GCCATAAA	2.842557
477	CCATAAAC	2.907907
478	CATAAACT	2.907907
479	ATAAACTG	2.834602

480	TAAACTGC(	2.904339
481	AAACTGCC	2.739062
482	AACTGCCA	2.724355
483	ACTGCCAG	2.720221
484	CTGCCAGC	2.723593
485	TGCCAGGC	2.706867
486	GCCAGGC/	2.711365
487	CCAGGCAT	2.647365
488	CAGGCATC	2.656858
489	AGGCATCA	2.541686
490	GGCATCAA	2.508998
491	GCATCAAA	2.428586
492	CATCAAAT	2.396968
493	ATCAAATT/	2.515454
494	TCAAATTA/	2.850679
495	CAAATTAA(	2.87819
496	AAATTAAG(	2.907039
497	AATTAAGC/	2.907039
498	ATTAAGCA(	2.931057
499	TTAAGCAG/	2.945752
500	TAAGCAGA	2.974586
501	AAGCAGAA	2.859101
502	AGCAGAAG	2.819107
503	GCAGAAGC	2.784013
504	CAGAAGGC	2.779349
505	AGAAGGCC	2.714623
506	GAAGGCC/	2.728977
507	AAGGCCAT	2.82497
508	AGGCCATC	2.856021
509	GGCCATCC	2.834426
510	GCCATCCT	2.834388
511	CCATCCTG	2.796763
512	CATCCTGA	2.739553
513	ATCCTGAC	2.777783
514	TCCTGACG	2.80293
515	CCTGACGC	2.87509
516	CTGACGG/	2.848768
517	TGACGGAT	2.808439
518	GACGGATG	2.914256
519	ACGGATGC	3.216129
520	CGGATGGC	3.245262
521	GGATGGCC	3.358862
522	GATGGCCT	3.373414
523	ATGGCCTT	3.387167
524	TGGCCTTT	3.425412
525	GGCCTTTT	3.397071
526	GCCTTTTC	3.425412
527	CCTTTTTGC	3.379815

528	CTTTTTGCC	3.319704
529	TTTTTGCGT	3.333368
530	TTTTGCGTT	3.470384
531	TTTGCGTTT	3.594772
532	TTGCGTTTC	3.719161
533	TGCGTTTCT	3.774124
534	GCGTTTCTA	3.745929
535	CGTTTCTAC	3.794416
536	GTTTCTACA	3.824347
537	TTTCTACAA	3.83425
538	TTCTACAAA	3.826609
539	TCTACAAAC	3.831538
540	CTACAAAC	3.929182
541	TACAAACTC	3.857858
542	ACAAACTC	3.589108
543	CAAACTCT	3.767565
544	AAACTCTTC	3.61876
545	AACTCTTCC	3.834341
546	ACTCTTCCT	3.832286
547	CTCTTCCTC	3.839791
548	TCTTCCTGT	3.7993
549	CTTCCTGTC	3.837659
550	TTCCTGTCC	4.033417
551	TCCTGTCG	4.12822
552	CCTGTCGT	4.118817
553	CTGTCGTC	4.167978
554	TGTCGTCAT	4.178889
555	GTCGTCATA	4.179198
556	TCGTCATAT	4.229561
557	CGTCATATC	4.255148
558	GTCATATCT	4.189178
559	TCATATCTA	4.205947
560	CATATCTAC	4.197337
561	ATATCTACA	4.062881
562	TATCTACAA	4.152682
563	ATCTACAAC	4.129279
564	TCTACAAG	4.120525
565	CTACAAGC	4.09929
566	TACAAGCC	3.979753
567	ACAAGCCA	3.667844
568	CAAGCCAT	3.692302
569	AAGCCATC	3.557951
570	AGCCATCC	3.538145
571	GCCATCCC	3.504091
572	CCATCCCC	3.477959
573	CATCCCCC	3.435925
574	ATCCCCCC	3.369334
575	TCCCCCCA	3.479783

576	CCCCCCA	3.492219
577	CCCCCAC	3.479232
578	CCCCACA	3.343532
579	CCCACAG	3.242928
580	CCACAGAT	3.114677
581	CACAGATA	3.085563
582	ACAGATAC	3.066264
583	CAGATACG	3.172303
584	AGATACGG	3.13288
585	GATACGGT	3.139222
586	ATACGGTA	3.21804
587	TACGGTAA	3.256448
588	ACGGTAAA	3.36307
589	CGGTAAAC	3.338289
590	GGTAAACT	3.280055
591	GTAAACTA	3.074087
592	TAAACTAG	3.199951
593	AAACTAGC	2.974847
594	AACTAGCC	3.066928
595	ACTAGCCT	3.091742
596	CTAGCCTC	3.086282
597	TAGCCTCG	3.011311
598	AGCCTCGT	2.943335
599	GCCTCGTT	2.860248
600	CCTCGTTT	2.995542
601	CTCGTTTT	3.008892
